# Synthesis, structure and computational study of *N*-acetyl*-N*′-(4-chloro­phen­yl)­thio­urea

**DOI:** 10.1107/S2056989026008315

**Published:** 2026-08-20

**Authors:** Sharatha Kumar, Tuncer Hökelek

**Affiliations:** a Department of Chemistry Yenepoya Institute of Arts Science Commerce and Management Mangaluru Yenepoya University (Deemed to be university) 575013 Karnataka India; b Hacettepe University, Department of Physics, 06800 Beytepe-Ankara, Türkiye; Universidad de la Repüblica, Uruguay

**Keywords:** acet­yl, thio­urea, 4-chloro­phen­yl, crystal structure

## Abstract

The title compound, C_9_H_9_ClN_2_OS, consists of a chloro­phenyl ring and acetyl moiety bridged over a thio­urea functional group. The dihedral angle between the acetyl group and the planar thio­urea group is 1.9 (5)°, and they are oriented at dihedral angles of 57.9 (5) and 59.77 (16)°, respectively, with respect to the phenyl ring. An intra­molecular N—H⋯O hydrogen bond forms an *S*(6) ring motif. In the crystal, N—H⋯S and N—H⋯O hydrogen bonds link the mol­ecules, enclosing *R*^2^_2_(8) and *R*^2^_2_(12) ring motifs, into infinite chains along the *a*-axis direction. Hirshfeld surface analysis revealed that the most important contributions for crystal packing are H⋯H (31.1%), H⋯Cl/Cl⋯H (16.9%), H⋯S/S⋯H (14.4%), H⋯C/C⋯H (12.9%) and H⋯O/O⋯H (9.4%) inter­actions. Computational methods revealed N—H⋯S and N—H⋯O hydrogen-bonding energies of −13.8 and −10.1 kJ mol^−1^, respectively. Evaluations of the electrostatic, dispersion and total energy frameworks indicate that the crystal stabilization is dominated by dispersion energy contributions.

## Abstract 2

The title compound consists of a chloro­phenyl ring and acetyl moiety bridged over a thio­urea functional group. In the crystal, N—H⋯S and N—H⋯O hydrogen bonds link the mol­ecules into infinite chains along the *a*-axis direction. Neither π–π nor C—H⋯π(ring) inter­actions are observed.

## 1. Chemical context

Acyl thio­urea derivatives have attracted considerable attention owing to their biological activities. These compounds have been extensively investigated for their pharmacological potentials, including anti­cancer properties, where they inhibit cancer cell proliferation and include cytotoxic effects, as well as anti­microbial activities against a broad spectrum of bacterial and fungal pathogens (Cui *et al.*, 2017 ▸; Asghar *et al.*, 2018 ▸; Asegbeloyin *et al.*, 2018 ▸; Saeed *et al.*, 2024 ▸; Sakhare *et al.*, 2026 ▸). In particular, several thio­urea derivatives have demonstrated promising anti­cancer activities through mechanisms involving enzyme inhibition and inter­ference with tumour cell growth (Cui *et al.*, 2017 ▸; Mistry *et al.*, 2017 ▸; Ashgar *et al.*, 2018 ▸; Zaman *et al.*, 2024 ▸; Ramos *et al.*, 2026 ▸; Fayyaz *et al.*, 2026 ▸).

In addition to their biological relevance, acyl thio­ureas exhibit inter­esting conformational preferences and supra­molecular behaviours in the solid state. Crystal structures of several 3-acetyl-1-aryl­thio­urea derivatives have been reported (Gowda *et al.*, 2012*d* ▸; Kumar *et al.*, 2012*b* ▸; Shahwar *et al.*, 2012*b* ▸). These compounds typically adopt a pseudo-*anti* conformation with respect to the carbonyl (C=O) and thio­carbonyl (C=S) groups and are commonly feature intra­molecular N—H⋯O hydrogen bonds. In the crystal, N—H⋯S hydrogen bonds further contribute to the consolidation of the packing arrangement.

As part of our ongoing investigations into acyl thio­urea derivatives, we report herein the synthesis, the mol­ecular and crystal structures together with Hirshfeld surface (HS) and crystal void analyses and inter­action energy calculations and energy frameworks of the title compound (**I**).
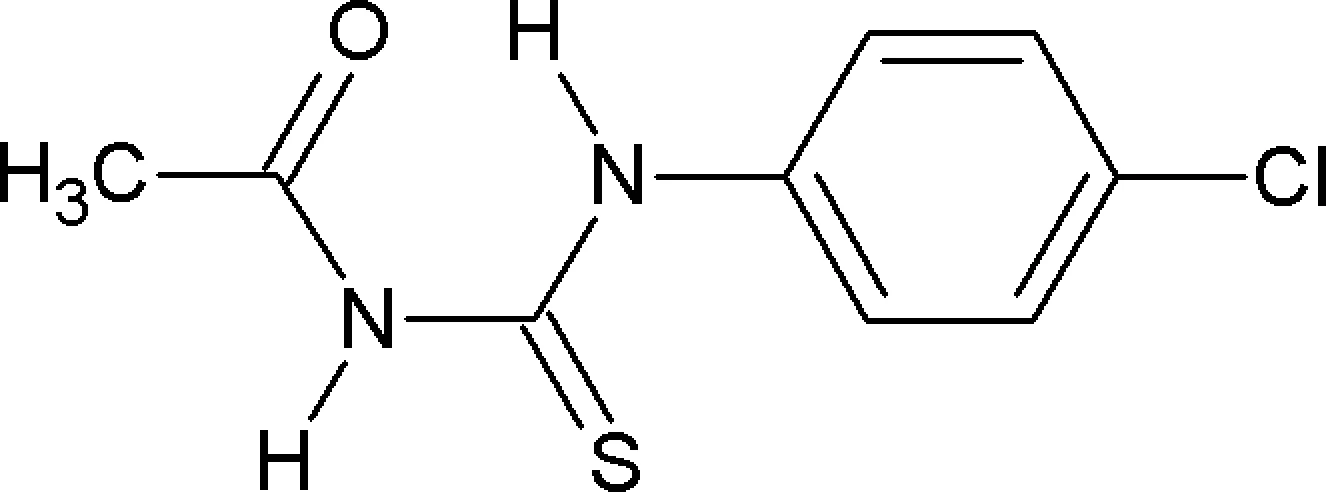


## 2. Structural commentary

**Figure 1:**
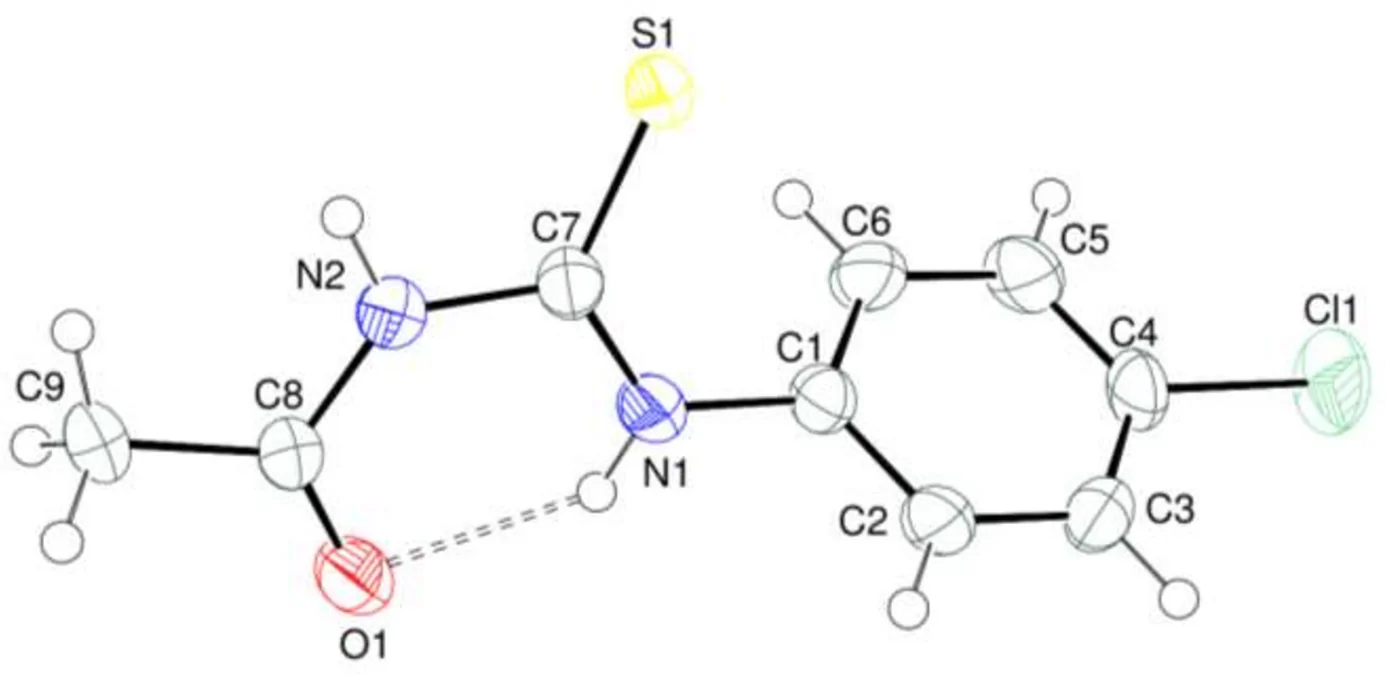
The mol­ecular structure with atom-numbering scheme and 50% probability ellipsoids. The intra­molecular N—H⋯O hydrogen bond is shown as a dashed line.

Compound (**I**) consists of a chloro­phenyl ring and an acetyl moiety bridged by the thio­urea functional group (Fig. 1 ▸). The two N—H bonds, beside the thio­carbonyl C=S and carbonyl C=O bonds are in *anti* conformations. The acetyl (O1/C8/C9) group is oriented at a dihedral angle of 1.9 (5)° with respect to the almost planar thio­urea (S1/C7/N1/N2) group (r.m.s. deviation = 0.005 Å). The dihedral angles between the phenyl (C1–C6) ring and the acetyl and thio­urea groups are 57.9 (5)° and 59.77 (16)°, respectively. The Cl1 and N1 atoms are −0.0638 (19) and 0.027 (5) Å away from the best plane of the phenyl ring. The bond lengths are in normal ranges (Allen *et al.*, 1987 ▸) and comparable to those in the similar compounds: *N*-(2-chloro­phen­yl)-*N*′-propano­ylthio­urea [(**II**); Kumar & Hökelek, 2026 ▸], *N*-(3-chloro­propion­yl)-*N*′-phenyl­thio­urea [(**III**); Othman *et al.*, 2010 ▸] and *N*-(2,6-di­methyl­phen­yl)-*N*′-propano­ylthio­urea [(**IV**); Yusof *et al.*, 2012 ▸], 3-acetyl-1-(2,6-di­methyl­phen­yl)thio­urea [(**V**); Kumar *et al.*, 2012*b* ▸], 3-acetyl-1-(2,5-di­methyl­phen­yl)thio­urea [(**VI**); Gowda *et al.*, 2012*d* ▸] and 3-acetyl-1-(2-methyl­phen­yl)thio­urea [(**VII**); Shahwar *et al.*, 2012*b* ▸]. The C1—N1—C7 [124.8 (5)°] and S1—C7—N1 [125.2 (5)°] bond angles in (**I**) are significantly wider than the corresponding values in (**III**) and (**IV**), while C1—N1—C7 and S1—C7—N1 bond angles are significantly narrower and the same, respectively, when compared with the corresponding values in (**II**). On the other hand, S1—C7—N1 and O1—C8—C9 [123.9 (6)°] bond angles in (**I**) are significantly wider than the corresponding values in (**V**), (**VI**) and (**VII**), while N2—C8—O1 [121.8 (6)°] and S1—C7—N2 [117.9 (5)°] bond angles are significantly narrower, when compared with the corresponding values in (**V**), (**VI**) and (**VII**). An intra­molecular N—H⋯O hydrogen bond (Table 1 ▸) in (**I**) forms an *S*(6) ring motif (Etter *et al.*, 1990 ▸) (Fig. 1 ▸).

## 3. Supra­molecular features

**Figure 2:**
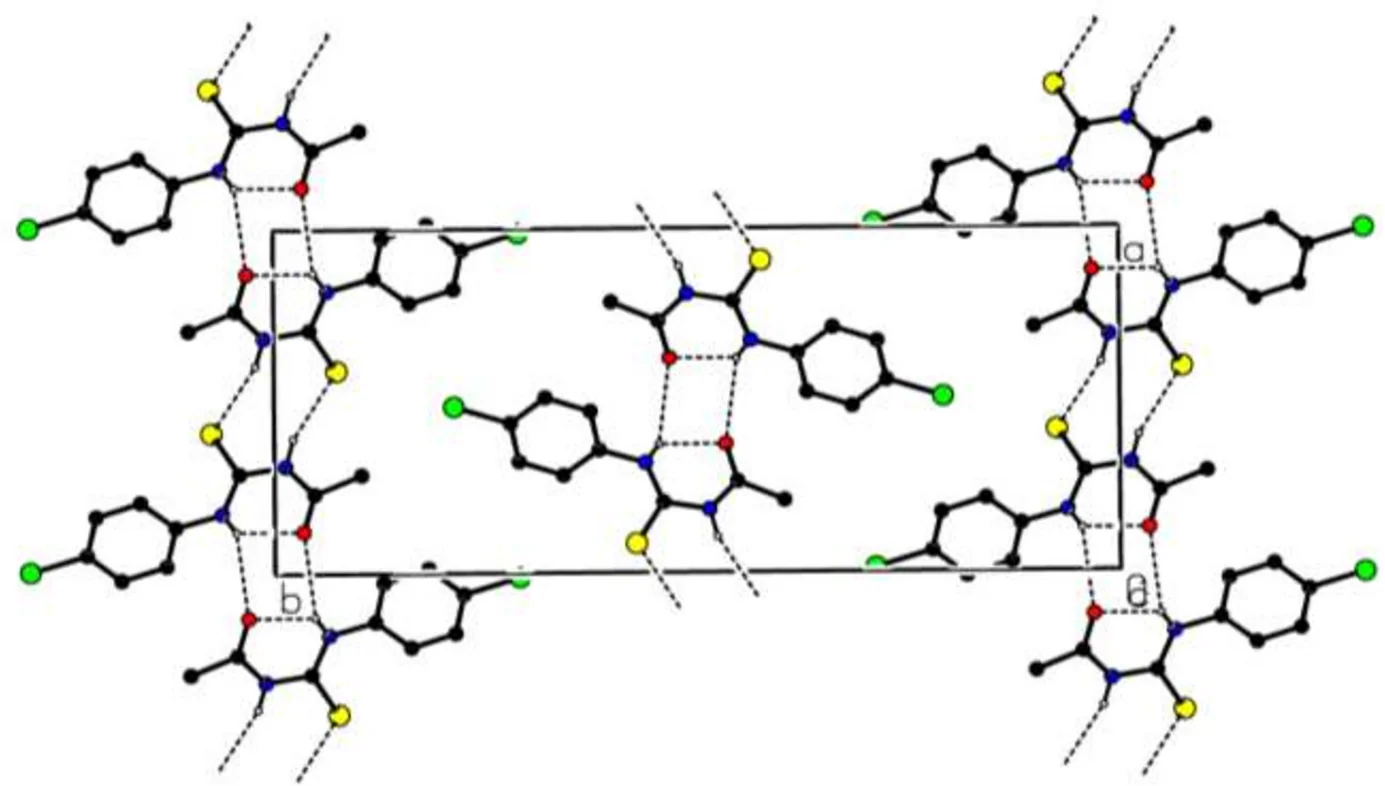
A partial packing diagram viewed down the *c*–axis direction showing the infinite chains along the *a*-axis direction. The intra­molecular N—H⋯O and inter­molecular N—H⋯S and N—H⋯O hydrogen bonds, which form S(6), 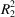
(8) and 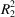
(12) ring motifs, are shown as dashed lines.

In the crystal, N—H⋯S and N—H⋯O hydrogen bonds (Table 1 ▸) link the mol­ecules, enclosing 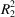
(8) and 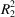
(12) ring motifs (Etter *et al.*, 1990 ▸), into infinite chains along the *a*-axis direction (Fig. 2 ▸). Neither π–π nor C—H⋯π(ring) inter­actions are observed.

**Figure 3:**
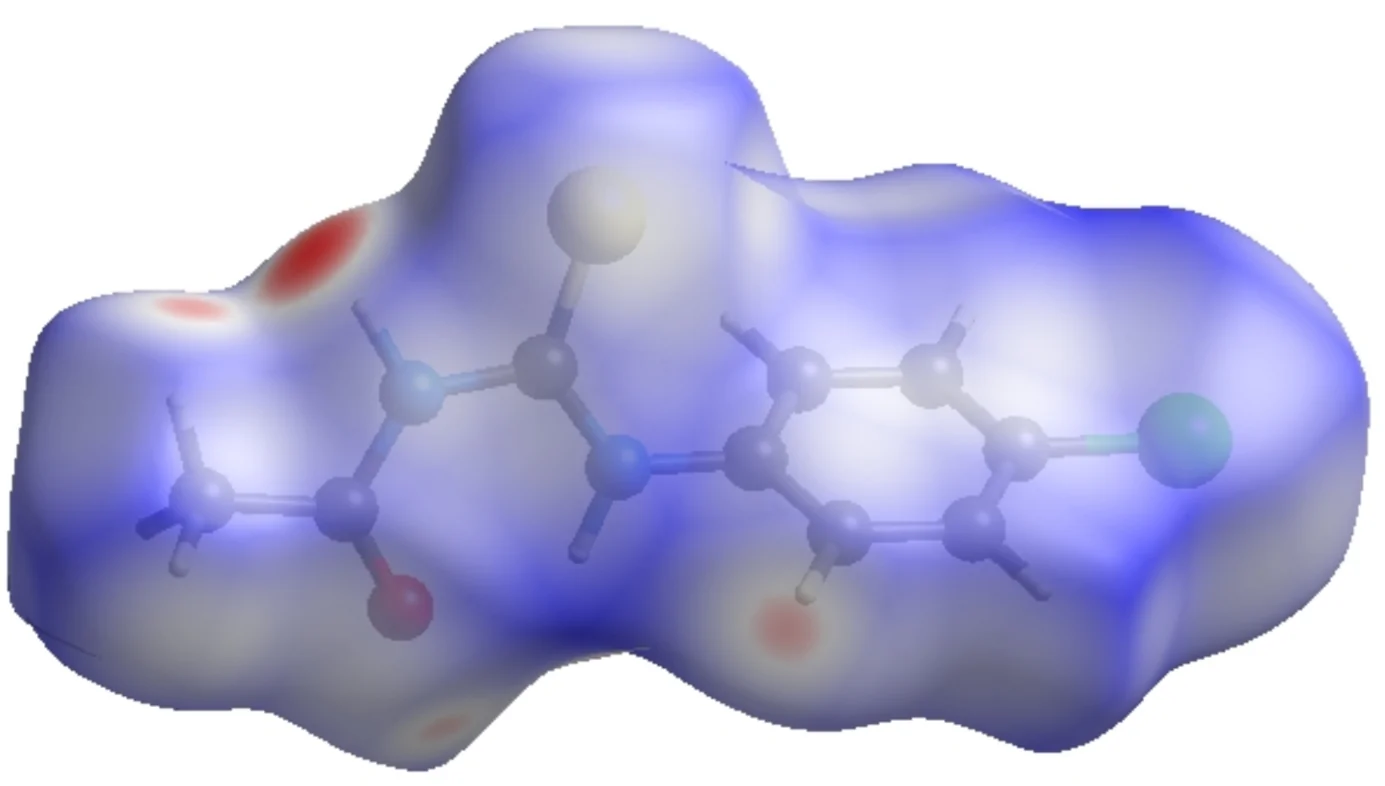
View of the three-dimensional Hirshfeld surface plotted over *d*_norm_ in the range −0.3415 to 1.2606 a.u.

**Figure 4:**
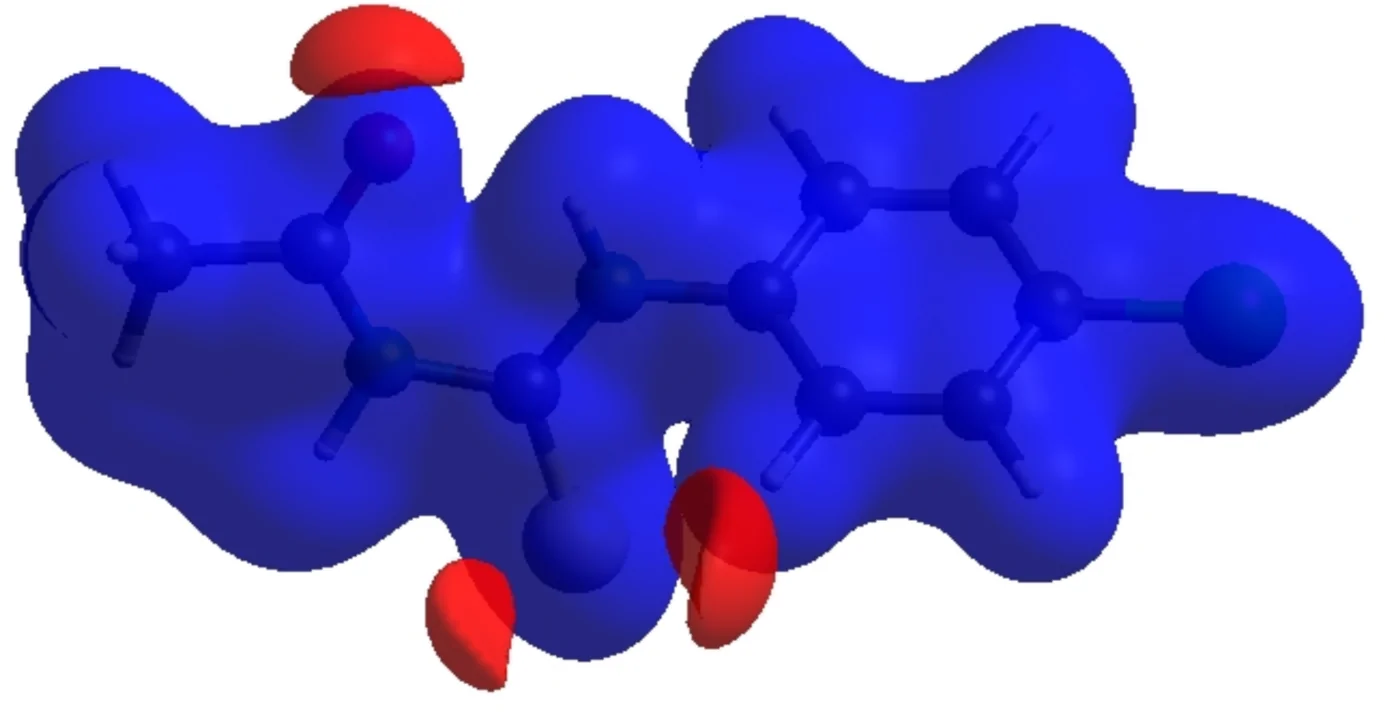
View of the three-dimensional Hirshfeld surface of the title compound plotted over electrostatic potential in the range of −0.0500 to 0.0500 a.u. using the STO-3 G basis set at the Hartree–Fock level of theory. Hydrogen-bond donors and acceptors are shown as blue and red regions around the atoms, corresponding to positive and negative potentials, respectively.

The inter­molecular inter­actions in the crystal were visualized by carrying out a Hirshfeld surface (HS) analysis using *CrystalExplorer* 17.5 (Spackman *et al.*, 2021 ▸). Fig. 3 ▸ shows the Hirshfeld surface mapped over *d*_norm_. The white surface indicates contacts with distances equal to the sum of van der Waals radii, and the red and blue colours indicate distances shorter (in close contact) or longer (distinct contacts) than the van der Waals radii, respectively. The red spots indicate their roles as the respective donor and/or acceptor atoms in hydrogen bonding. They also appear as the blue and red regions corresponding to positive and negative potentials on the HS mapped over electrostatic potential shown in Fig. 4 ▸. The blue and red regions indicate positive (hydrogen-bond donors) and negative (hydrogen-bond acceptors) electrostatic potentials.

**Figure 5:**
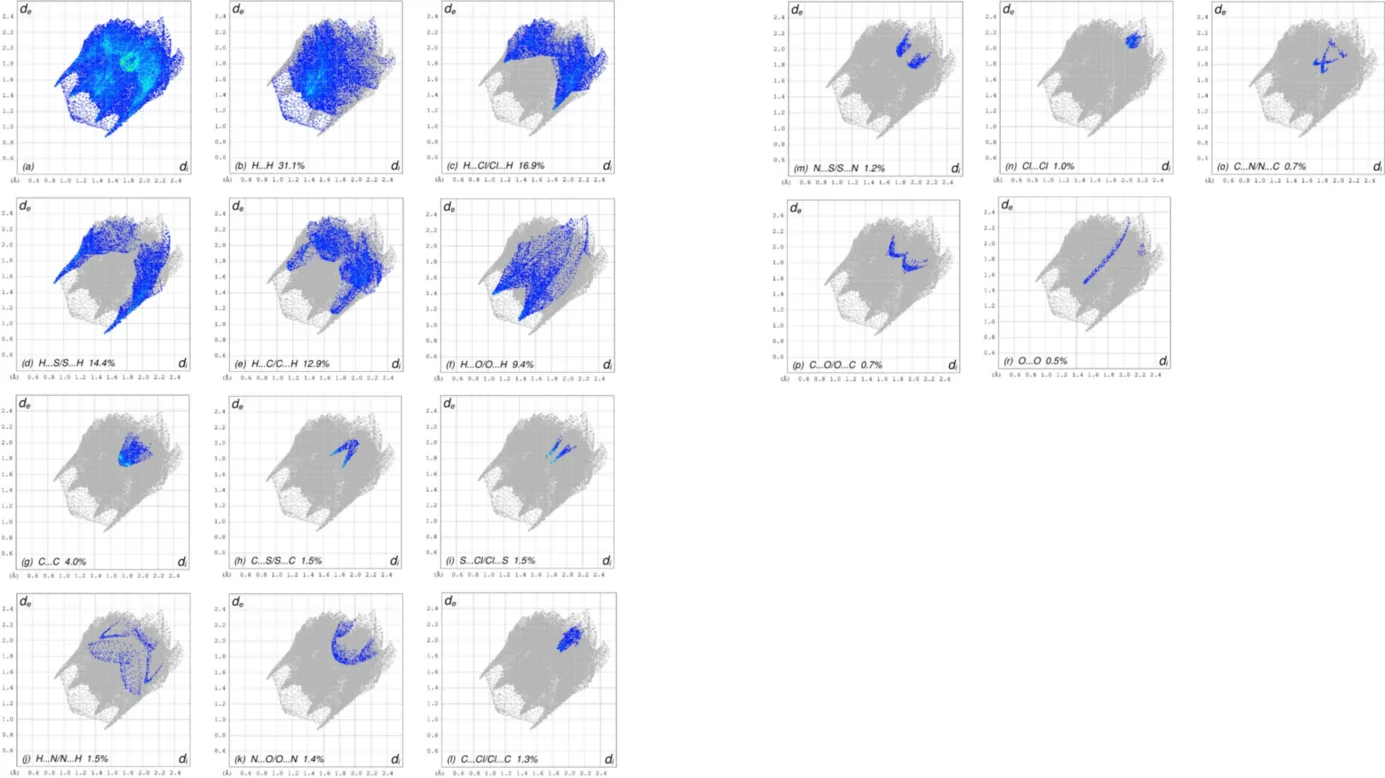
The full two-dimensional fingerprint plots for title mol­ecule, showing (*a*) all inter­actions, and delineated into (*b*) H⋯H, (*c*) H⋯Cl/Cl⋯H, (*d*) H⋯S/S⋯H, (*e*) H⋯C/C⋯H, (*f*) H⋯O/O⋯H, (*g*) C⋯C, (*h*) C⋯S/S⋯C, (*i*) S⋯Cl/Cl⋯S, (*j*) H⋯N/N⋯H, (*k*) N⋯O/O⋯N, (*l*) C⋯Cl/Cl⋯C, (*m*) N⋯S/S⋯N, (*n*) Cl⋯Cl, (*o*) C⋯N/N⋯C, (*p*) C⋯O/O⋯C and (*r*) O⋯O inter­actions. The *d*_i_ and *d*_e_ values are the closest inter­nal and external distances (in Å) from given points on the Hirshfeld surface.

The overall two-dimensional fingerprint plot is shown in Fig. 5 ▸*a* and those delineated into different contact types are illustrated in Fig. 5 ▸*b*–*r*, respectively. According to the two-dimensional fingerprint plots, the H⋯H, H⋯Cl/Cl⋯H, H⋯S/S⋯H, H⋯C/C⋯H and H⋯O/O⋯H contacts make the most significant contributions to the HS, at 31.1%, 16.9%, 14.4%, 12.9% and 9.4%, respectively (Fig. 5 ▸). In *N*-(2-chloro­phen­yl)-*N*′-propano­ylthio­urea [(**II**); Kumar & Hökelek, 2026 ▸], the most important contributions for the crystal packing are H⋯H (39.2%), H⋯Cl/Cl⋯H (15.8%), H⋯S/S⋯H (14.2%) and H⋯C/C⋯ H (9.9%) inter­actions.

**Figure 6:**
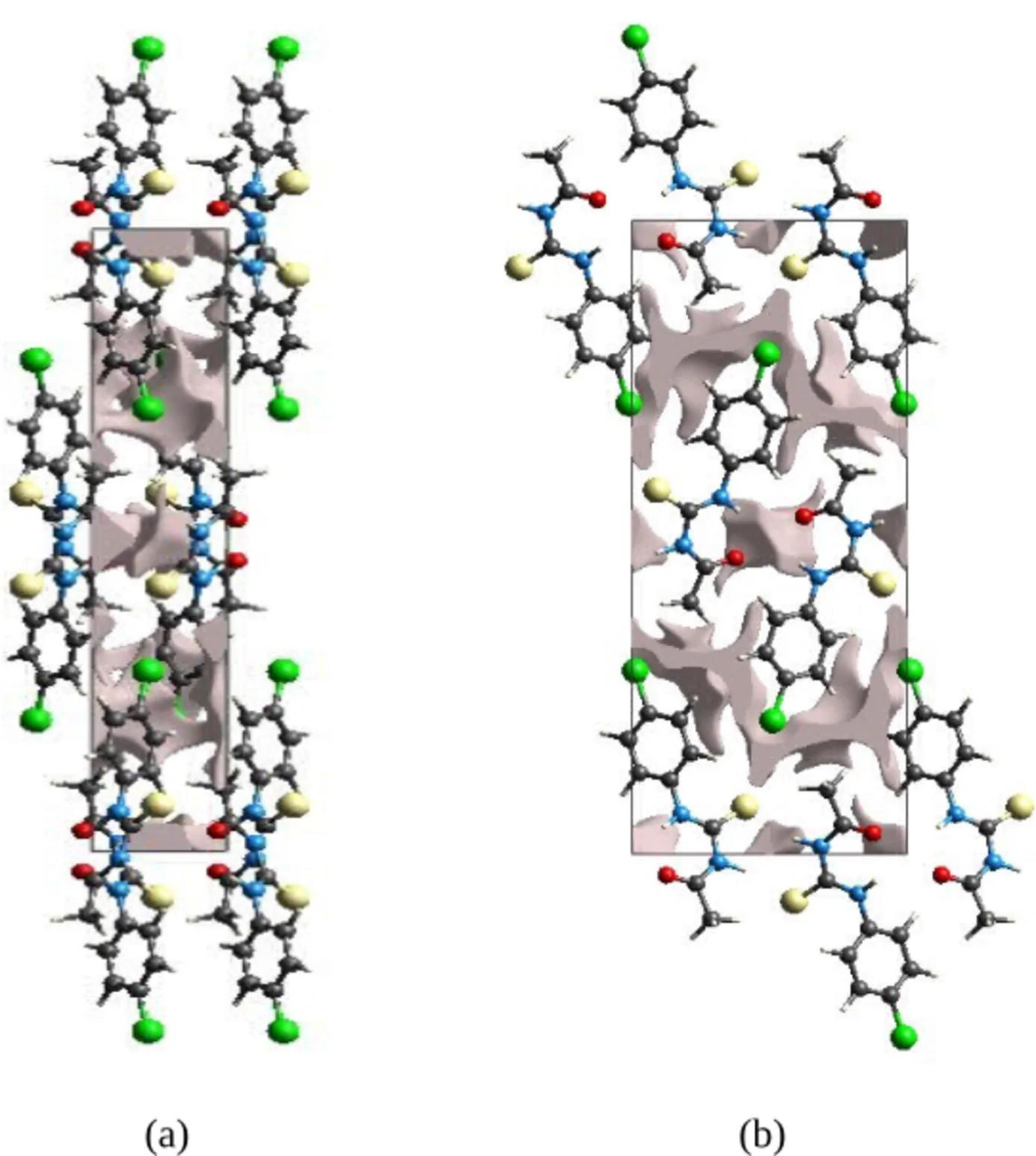
Crystal voids viewed down the (*a*) *a*-axis and (*b*) *c*-axis directions.

The volume of the crystal voids (Fig. 6 ▸*a*,*b*) and the percentage of free space in the unit cell were calculated to be 87.5 Å^3^ and 8.6%, respectively. Thus, the crystal packing appears compact and the mechanical stability should be substantial. In compound (**II**), the volume of the crystal voids and the percentage of free space in the unit cell were calculated to be 105.62 Å^3^ and 9.33%, respectively, showing that there was no large cavity in the crystal packing.

The inter­molecular inter­action energies were calculated using CE–B3LYP/6–31G(d,p) energy model available in *CrystalExplorer* 17.5 (Spackman *et al.*, 2021 ▸), where a cluster of mol­ecules is generated by applying crystallographic symmetry operations with respect to a selected central mol­ecule within the radius of 3.8 Å by default. The total inter­molecular energy (*E*_tot_) is the sum of electrostatic (*E*_ele_), polarization (*E*_pol_), dispersion (*E*_dis_) and exchange-repulsion (*E*_rep_) energies (Turner *et al.*, 2015 ▸) with scale factors of 1.057, 0.740, 0.871 and 0.618, respectively (Mackenzie *et al.*, 2017 ▸). Hydrogen-bonding inter­action energies (in kJ mol^−1^) were calculated to be −9.1 (*E*_ele_), −1.4 (*E*_pol_), −6.6 (*E*_dis_), 4.3 (E_rep_) and −13.8 (*E*_tot_) for the N2—H2*N*⋯S1 and −4.5 (*E*_ele_), −1.4 (*E*_pol_), −10.8 (*E*_dis_), 8.3 (*E*_rep_) and −10.1 (*E*_tot_) for the N1—H1*N⋯*O1 hydrogen-bonding inter­actions (see supporting information).

**Figure 7:**
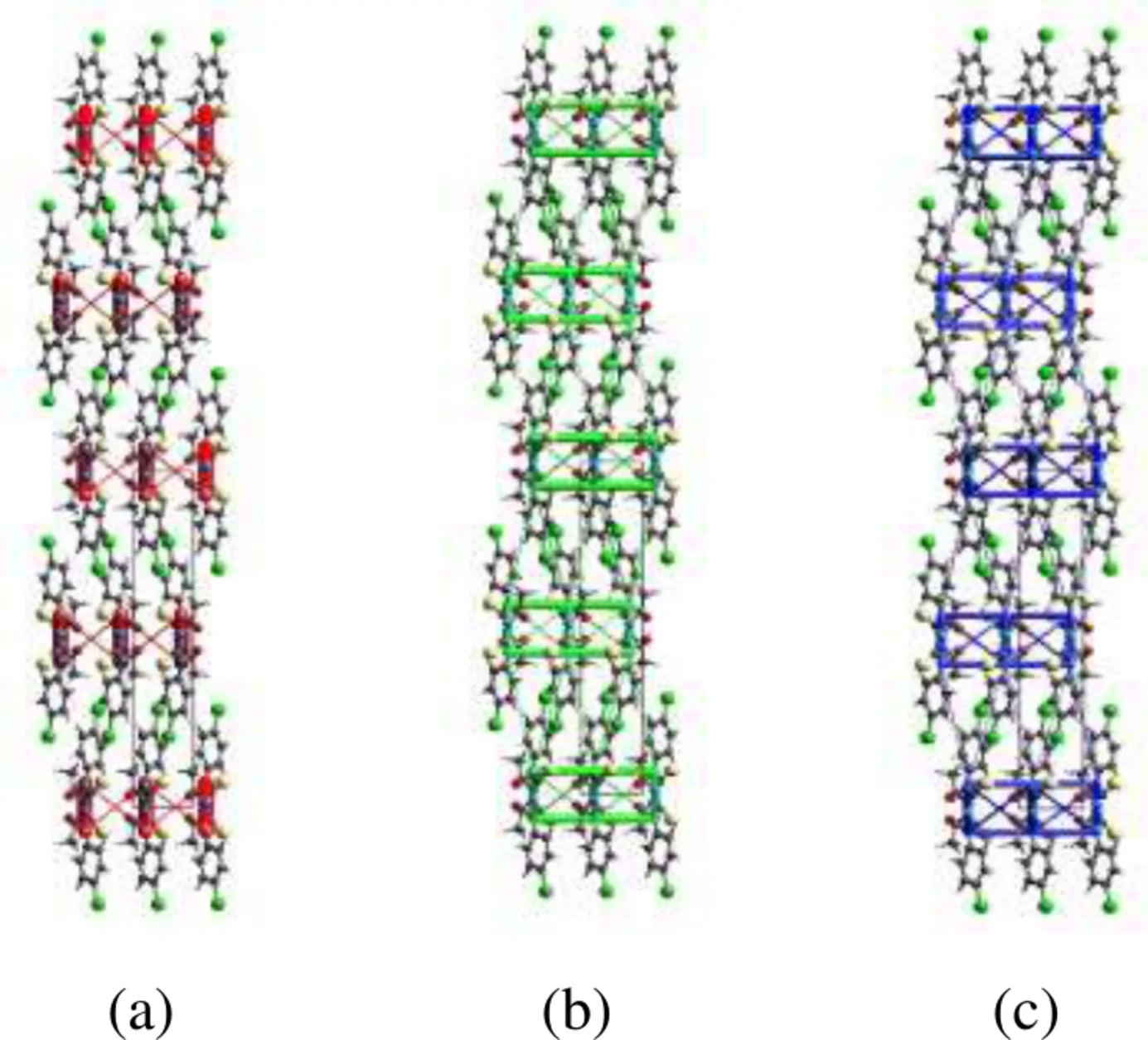
The energy frameworks for a cluster of mol­ecules, viewed down the *a* axis, showing the (*a*) electrostatic energy, (*b*) dispersion energy and (*c*) total energy diagrams. The cylindrical radius is proportional to the relative strength of the corresponding energies and they were adjusted to the same scale factor of 80 with cut-off value of 5 kJ mol^−1^ within 2 × 2 × 2 unit cells.

Energy frameworks combine the calculation of inter­molecular inter­action energies with a graphical representation of their magnitudes, and were constructed for *E*_ele_ (red cylinders), *E*_dis_ (green cylinders) and *E*_tot_ (blue cylinders) (Fig. 7 ▸*a*,*b*,*c*). Evaluation of the electrostatic, dispersion and total energy frameworks indicates that the stabilization of the crystal structure is dominated by the dispersion energy contributions.

## 4. Database survey

A search of the Cambridge Structural Database (CSD, Version 6.00, updated May 2025; Groom *et al.*, 2016 ▸) revealed eleven closely related structures of *N*-acetyl-*N*′-aryl­thio­urea derivatives: 3-acetyl-1-phenyl­thio­urea, C_9_H_10_N_2_OS (CSD refcode GARBIM; Shahwar *et al.*, 2012*a* ▸), 3-acetyl-1-(2-methyl­phen­yl)thio­urea, C_10_H_12_N_2_OS (CSD refcode YAXNUI; Shahwar *et al.*, 2012*b* ▸), 3-acetyl-1-(3-chloro­phen­yl)thio­urea, C_9_H_9_ClN_2_OS (YAXTIC; Shahwar *et al.*, 2012*c* ▸), 3-acetyl-1-(3-methyl­phen­yl)thio­urea, C_10_H_12_N_2_OS (LEGQEV; Gowda *et al.*, 2012*a* ▸), 3-acetyl-1-(4-methyl­phen­yl)thio­urea, C_10_H_12_N_2_OS (XAZYOO; Gowda *et al.*, 2012*b* ▸), 3-acetyl-1-(2,4-di­methyl­phen­yl)thio­urea, C_11_H_14_N_2_OS (LEGCIL; Gowda *et al.*, 2012*c* ▸), 3-acetyl-1-(2,5-di­methyl­phen­yl)thio­urea, C_11_H_14_N_2_OS (LEDYAW; Gowda *et al.*, 2012*d* ▸), 3-acetyl-1-(2,3-di­chloro­phen­yl)thio­urea, C_9_H_8_Cl_2_N_2_OS (LEFBEF; Gowda *et al.*, 2012*e* ▸), 3-acetyl-1-(2,3-di­methyl­phen­yl)thio­urea, C_11_H_14_N_2_OS (XEBKUM; Kumar *et al.*, 2012*a* ▸), 3-acetyl-1-(2,6-di­methyl­phen­yl)thio­urea, C_11_H_14_N_2_OS (Kumar *et al.*, 2012*b* ▸), 3-acetyl-1-(2,6-di­chloro­phen­yl)thio­urea, C_9_H_8_Cl_2_N_2_OS (CLEDSUK; Kumar *et al.*, 2012*c* ▸). Overall, these *N*-acetyl-*N*′-aryl­thio­urea derivatives consistently exhibit hydrogen-bonding networks dominated by N—H⋯S and N—H⋯O inter­actions, which frequently generate graph-set motifs such as 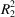
(8) and 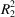
(12), and assemble the mol­ecules into one-, two- or three-dimensional supra­molecular architectures. In several structures, three-centre N—H⋯(O,S) hydrogen bonds and weaker C—H⋯S contacts further reinforce the crystal packing, highlighting the key role of hydrogen bonding in consolidating this family of compounds.

## 5. Synthesis and crystallization

The title compound was synthesized by adding a solution of acetyl chloride (0.10 mol) in acetone (30 ml) dropwise to a suspension of potassium thio­cyanate (0.10 mol) in acetone (30 ml), and then stirred for 2 h. Then, the reaction mixture was refluxed for 30 min. After cooling to room temperature, a solution of 4-chloro­aniline (0.10 mol) in acetone (10 ml) was added and the solution refluxed for 3 h. After completion of the reaction (monitored by TLC), the reaction mixture was poured into acidified cold water. The solids separated were filtered under suction, washed thoroughly with water and dried under vacuum. Colourless crystals suitable for X-ray analysis were obtained by slow evaporation of an aceto­nitrile solution. White solid, yield 85%, m.p. 445 K. IR (KBr) cm^−1^: 3181 (N—H str.), 1164 (C=S str.) and 1690 (C=O str.).

## 6. Refinement

Crystal data, data collection and structure refinement details are summarized in Table 2 ▸. The NH hydrogen atoms were located from a difference-Fourier map and refined isotropically. The C-bound hydrogen-atom positions were calculated geometrically at distances of 0.93 Å (for aromatic CH) and 0.96 Å (for methyl CH) and refined using a riding model with U_iso_(H) = *k* × U_eq_ (C, N), where *k* = 1.5 for methyl hydrogens and *k* = 1.2 for other H atoms.

## Supplementary Material

Crystal structure: contains datablock(s) I, global. DOI: 10.1107/S2056989026008315/ny2021sup1.cif

Structure factors: contains datablock(s) I. DOI: 10.1107/S2056989026008315/ny2021Isup2.hkl

Interaction energies. DOI: 10.1107/S2056989026008315/ny2021sup3.pdf

CCDC reference: 1816806

Additional supporting information:  crystallographic information; 3D view; checkCIF report

## Figures and Tables

**Table 1:** Hydrogen-bond geometry (Å, °)

*D*—H⋯*A*	*D*—H	H⋯*A*	*D*⋯*A*	*D*—H⋯*A*
N1—H1*N*⋯O1	0.88 (3)	2.04 (6)	2.662 (7)	128 (6)
N1—H1*N*⋯O1^i^	0.88 (3)	2.59 (5)	3.319 (7)	141 (6)
N2—H2*N*⋯S1^ii^	0.87 (3)	2.54 (3)	3.379 (6)	163 (6)

Symmetry codes: (i) 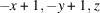; (ii) 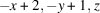.

**Table 2:** Experimental details

Crystal data	
Chemical formula	C_9_H_9_ClN_2_OS
*M*_r_	228.69
Crystal system, space group	Orthorhombic, *P*2_1_2_1_2
Temperature (K)	293
*a*, *b*, *c* (Å)	10.090 (3), 24.830 (6), 4.079 (2)
*V* (Å^3^)	1021.9 (6)
*Z*	4
Radiation type	Mo *K*α
μ (mm^−1^)	0.54
Crystal size (mm)	0.50 × 0.08 × 0.06

Data collection	
Diffractometer	Oxford Diffraction Xcalibur with Sapphire CCD
Absorption correction	Multi-scan (*CrysAlis RED*; Oxford Diffraction, 2009 ▸)
*T*_min_, *T*_max_	0.772, 0.968
No. of measured, independent and observed [*I* > 2σ(*I*)] reflections	2960, 1803, 1527
*R*_int_	0.018
(sin θ/λ)_max_ (Å^−1^)	0.613

Refinement	
*R*[*F*^2^ > 2σ(*F*^2^)], *wR*(*F*^2^), *S*	0.060, 0.152, 1.26
No. of reflections	1803
No. of parameters	134
No. of restraints	2
H-atom treatment	H atoms treated by a mixture of independent and constrained refinement
Δρ_max_, Δρ_min_ (e Å^−3^)	0.63, −0.28
Absolute structure	Flack *x* determined using 495 quotients [(*I*^+^)−(*I*^−^)]/[(*I*^+^)+(*I*^−^)] (Parsons et al., 2013 ▸), 672 Friedel pairs
Absolute structure parameter	0.54 (5)

Computer programs: *CrysAlis CCD* and *CrysAlis RED* (Oxford Diffraction, 2009 ▸), *SHELXT2013/1* (Sheldrick, 2015*a* ▸), *SHELXL2014/6* (Sheldrick, 2015*b* ▸), *ORTEP-3 for Windows* and *WinGX* publication routines (Farrugia, 2012 ▸) and *PLATON* (Spek, 2020 ▸).

## supplementary crystallographic information

### N-Acetyl-N'-(4-chlorophenyl)thiourea Crystal data

**Table:**

C_9_H_9_ClN_2_OS	*D*_x_ = 1.486 Mg m^−^^3^
*M**_r_* = 228.69	Mo *K*α radiation, λ = 0.71073 Å
Orthorhombic, *P*2_1_2_1_2	Cell parameters from 1233 reflections
*a* = 10.090 (3) Å	θ = 3.2–28.0°
*b* = 24.830 (6) Å	µ = 0.54 mm^−^^1^
*c* = 4.079 (2) Å	*T* = 293 K
*V* = 1021.9 (6) Å^3^	Needle, colourless
*Z* = 4	0.50 × 0.08 × 0.06 mm
*F*(000) = 472	

### N-Acetyl-N'-(4-chlorophenyl)thiourea Data collection

**Table:**

Oxford Diffraction Xcalibur with Sapphire CCD diffractometer	1527 reflections with *I* > 2σ(*I*)
Rotation method data acquisition using ω scans.	*R*_int_ = 0.018
Absorption correction: multi-scan (CrysAlis RED; Oxford Diffraction, 2009)	θ_max_ = 25.8°, θ_min_ = 2.6°
*T*_min_ = 0.772, *T*_max_ = 0.968	*h* = −12→7
2960 measured reflections	*k* = −30→27
1803 independent reflections	*l* = −4→4

### N-Acetyl-N'-(4-chlorophenyl)thiourea Refinement

**Table:**

Refinement on *F*^2^	Hydrogen site location: mixed
Least-squares matrix: full	H atoms treated by a mixture of independent and constrained refinement
*R*[*F*^2^ > 2σ(*F*^2^)] = 0.060	*w* = 1/[σ^2^(*F*_o_^2^) + (0.0725*P*)^2^ + 0.5752*P*] where *P* = (*F*_o_^2^ + 2*F*_c_^2^)/3
*wR*(*F*^2^) = 0.152	(Δ/σ)_max_ < 0.001
*S* = 1.26	Δρ_max_ = 0.63 e Å^−^^3^
1803 reflections	Δρ_min_ = −0.28 e Å^−^^3^
134 parameters	Absolute structure: Flack *x* determined using 495 quotients [(*I*^+^)-(*I*^-^)]/[(*I*^+^)+(*I*^-^)] (Parsons et al., 2013), 672 Friedel pairs.
2 restraints	Absolute structure parameter: 0.54 (5)

### N-Acetyl-N'-(4-chlorophenyl)thiourea Special details

**Table:**

Geometry. All esds (except the esd in the dihedral angle between two l.s. planes) are estimated using the full covariance matrix. The cell esds are taken into account individually in the estimation of esds in distances, angles and torsion angles; correlations between esds in cell parameters are only used when they are defined by crystal symmetry. An approximate (isotropic) treatment of cell esds is used for estimating esds involving l.s. planes.

### N-Acetyl-N'-(4-chlorophenyl)thiourea Fractional atomic coordinates and isotropic or equivalent isotropic displacement parameters (Å^2^)

**Table:**

	*x*	*y*	*z*	*U*_iso_*/*U*_eq_	
C1	0.6395 (6)	0.3823 (2)	0.2485 (17)	0.0330 (15)	
C2	0.5266 (6)	0.3718 (2)	0.4246 (18)	0.0393 (18)	
H2	0.4776	0.3999	0.5148	0.047*	
C3	0.4861 (6)	0.3184 (2)	0.4666 (19)	0.0415 (15)	
H3	0.4082	0.3109	0.5795	0.050*	
C4	0.5605 (7)	0.2774 (2)	0.3427 (19)	0.0416 (18)	
C5	0.6731 (7)	0.2879 (3)	0.166 (2)	0.0462 (18)	
H5	0.7223	0.2597	0.0787	0.055*	
C6	0.7133 (7)	0.3404 (3)	0.118 (2)	0.046 (2)	
H6	0.7896	0.3477	−0.0020	0.055*	
C7	0.7919 (6)	0.4582 (2)	0.2858 (17)	0.0320 (15)	
C8	0.7334 (6)	0.5465 (2)	0.023 (2)	0.0386 (16)	
C9	0.7889 (6)	0.6019 (2)	−0.022 (2)	0.0418 (17)	
H9A	0.8744	0.6041	0.0801	0.063*	
H9B	0.7304	0.6278	0.0766	0.063*	
H9C	0.7973	0.6095	−0.2519	0.063*	
N1	0.6773 (5)	0.4373 (2)	0.1981 (16)	0.0390 (14)	
H1N	0.625 (5)	0.454 (2)	0.060 (14)	0.047*	
N2	0.8134 (5)	0.5127 (2)	0.2074 (16)	0.0379 (14)	
H2N	0.893 (4)	0.522 (2)	0.260 (18)	0.045*	
O1	0.6252 (4)	0.53307 (18)	−0.0660 (16)	0.0561 (16)	
Cl1	0.5120 (2)	0.21064 (7)	0.4144 (6)	0.0678 (8)	
S1	0.91037 (14)	0.42582 (6)	0.4885 (5)	0.0383 (4)	

### N-Acetyl-N'-(4-chlorophenyl)thiourea Atomic displacement parameters (Å^2^)

**Table:**

	*U*^11^	*U*^22^	*U*^33^	*U*^12^	*U*^13^	*U*^23^
C1	0.032 (3)	0.027 (3)	0.039 (4)	−0.003 (2)	−0.006 (3)	0.001 (3)
C2	0.035 (3)	0.026 (3)	0.057 (5)	0.004 (3)	0.003 (3)	−0.001 (3)
C3	0.036 (3)	0.037 (3)	0.051 (5)	−0.003 (3)	0.010 (4)	0.004 (3)
C4	0.046 (4)	0.024 (3)	0.055 (5)	−0.008 (3)	−0.006 (3)	0.008 (3)
C5	0.048 (4)	0.027 (3)	0.063 (5)	0.007 (3)	0.001 (3)	−0.005 (3)
C6	0.033 (4)	0.034 (3)	0.070 (6)	0.001 (3)	0.005 (3)	−0.001 (3)
C7	0.033 (3)	0.029 (3)	0.034 (4)	0.000 (3)	0.002 (3)	0.003 (3)
C8	0.033 (3)	0.027 (3)	0.055 (5)	0.000 (2)	0.004 (4)	0.007 (3)
C9	0.044 (4)	0.024 (3)	0.058 (5)	0.002 (3)	−0.001 (4)	0.007 (3)
N1	0.032 (3)	0.028 (3)	0.057 (4)	0.001 (2)	−0.005 (3)	0.006 (2)
N2	0.030 (3)	0.030 (3)	0.053 (4)	−0.002 (2)	−0.002 (3)	0.004 (2)
O1	0.040 (3)	0.035 (2)	0.093 (5)	−0.002 (2)	−0.019 (3)	0.012 (3)
Cl1	0.0788 (14)	0.0269 (8)	0.098 (2)	−0.0128 (9)	0.0097 (12)	0.0051 (9)
S1	0.0337 (7)	0.0301 (7)	0.0511 (11)	−0.0010 (6)	−0.0045 (9)	0.0102 (8)

### N-Acetyl-N'-(4-chlorophenyl)thiourea Geometric parameters (Å, º)

**Table:**

C1—C2	1.372 (9)	C7—N1	1.317 (8)
C1—C6	1.386 (9)	C7—N2	1.406 (8)
C1—N1	1.433 (8)	C7—S1	1.661 (6)
C2—C3	1.398 (8)	C8—O1	1.198 (7)
C2—H2	0.9300	C8—N2	1.385 (9)
C3—C4	1.363 (9)	C8—C9	1.498 (8)
C3—H3	0.9300	C9—H9A	0.9600
C4—C5	1.370 (10)	C9—H9B	0.9600
C4—Cl1	1.753 (6)	C9—H9C	0.9600
C5—C6	1.380 (9)	N1—H1N	0.88 (3)
C5—H5	0.9300	N2—H2N	0.87 (3)
C6—H6	0.9300		
			
C2—C1—C6	120.3 (6)	N1—C7—N2	116.9 (6)
C2—C1—N1	118.5 (6)	N1—C7—S1	125.2 (5)
C6—C1—N1	121.1 (6)	N2—C7—S1	117.9 (5)
C1—C2—C3	119.1 (6)	O1—C8—N2	121.8 (6)
C1—C2—H2	120.4	O1—C8—C9	123.9 (6)
C3—C2—H2	120.4	N2—C8—C9	114.0 (6)
C4—C3—C2	120.2 (6)	C8—C9—H9A	109.5
C4—C3—H3	119.9	C8—C9—H9B	109.5
C2—C3—H3	119.9	H9A—C9—H9B	109.5
C3—C4—C5	120.6 (6)	C8—C9—H9C	109.5
C3—C4—Cl1	119.4 (6)	H9A—C9—H9C	109.5
C5—C4—Cl1	120.0 (5)	H9B—C9—H9C	109.5
C4—C5—C6	119.9 (6)	C7—N1—C1	124.8 (5)
C4—C5—H5	120.1	C7—N1—H1N	122 (4)
C6—C5—H5	120.1	C1—N1—H1N	112 (4)
C5—C6—C1	119.8 (7)	C8—N2—C7	128.0 (6)
C5—C6—H6	120.1	C8—N2—H2N	121 (4)
C1—C6—H6	120.1	C7—N2—H2N	110 (4)
			
C6—C1—C2—C3	−0.8 (10)	N1—C1—C6—C5	−178.9 (6)
N1—C1—C2—C3	178.0 (6)	N2—C7—N1—C1	178.1 (6)
C1—C2—C3—C4	2.1 (11)	S1—C7—N1—C1	−3.6 (10)
C2—C3—C4—C5	−2.3 (12)	C2—C1—N1—C7	122.7 (7)
C2—C3—C4—Cl1	177.1 (5)	C6—C1—N1—C7	−58.6 (10)
C3—C4—C5—C6	1.2 (12)	O1—C8—N2—C7	7.8 (13)
Cl1—C4—C5—C6	−178.1 (6)	C9—C8—N2—C7	−178.0 (7)
C4—C5—C6—C1	0.0 (11)	N1—C7—N2—C8	−6.1 (11)
C2—C1—C6—C5	−0.2 (11)	S1—C7—N2—C8	175.5 (6)

### N-Acetyl-N'-(4-chlorophenyl)thiourea Hydrogen-bond geometry (Å, º)

**Table:**

*D*—H···*A*	*D*—H	H···*A*	*D*···*A*	*D*—H···*A*
N1—H1*N*···O1	0.88 (3)	2.04 (6)	2.662 (7)	128 (6)
N1—H1*N*···O1^i^	0.88 (3)	2.59 (5)	3.319 (7)	141 (6)
N2—H2*N*···S1^ii^	0.87 (3)	2.54 (3)	3.379 (6)	163 (6)

Symmetry codes: (i) −*x*+1, −*y*+1, *z*; (ii) −*x*+2, −*y*+1, *z*.
