# Supplementary material for: Synthesis, structure and computational study of N-acetyl-N′-(4-chloro­phen­yl)­thio­urea
Source: Acta Crystallogr E Crystallogr Commun. 2026 Aug 20;82(Pt 9):1066–70. doi: 10.1107/S2056989026008315 (PMC13548751; doi:10.1107/S2056989026008315)
Supplement: Supplementary file 1 [file e-82-01066-sup3.pdf]

	N	Symop	R	Electron Density	E_ele	E_pol	E_dis	E_rep	E_tot
	2	x, y, z	4.08	B3LYP/6-31G(d,p)	-4.6	-4.8	-51.0	36.3	-30.4
	2	-x, -y, z	7.58	B3LYP/6-31G(d,p)	-4.5	-1.4	-10.8	8.3	-10.1
	2	x+1/2, -y+1/2, -z	9.03	B3LYP/6-31G(d,p)	-2.0	-0.4	-6.6	3.3	-6.1
	2	-x+1/2, y+1/2, -z	12.68	B3LYP/6-31G(d,p)	-2.2	-0.4	-4.7	3.4	-4.6
	1	-x, -y, z	6.39	B3LYP/6-31G(d,p)	-14.3	-3.2	-31.1	23.0	-30.3
	2	x+1/2, -y+1/2, -z	8.97	B3LYP/6-31G(d,p)	-1.6	-0.9	-10.6	10.3	-5.2
	2	-x+1/2, y+1/2, -z	12.64	B3LYP/6-31G(d,p)	-2.2	-0.3	-2.6	0.8	-4.3
	1	-x, -y, z	8.31	B3LYP/6-31G(d,p)	-63.5	-15.4	-23.7	102.0	-36.2
	2	-x, -y, z	9.26	B3LYP/6-31G(d,p)	-9.1	-1.4	-6.6	4.3	-13.8
